# The bridge between anxiety and insomnia symptoms among Chinese adults before and after SARS-CoV-2 vaccination: a network analysis

**DOI:** 10.3389/fpsyt.2025.1604309

**Published:** 2025-07-14

**Authors:** Yan Liu, Shangyu Luo, Chao Jiang, Jing Guo, Minhui Dai, Li Feng, Mingxia Li, Jun Wen, Xiaobo Zhang

**Affiliations:** ^1^ Department of Critical Care Medicine, The Second People`s Hospital of Shenzhen & First Affiliated Hospital of Shenzhen University, Health Science Center, Shenzhen, China; ^2^ Department of Nosocomial Infection Prevention and Control, Shenzhen Second People’s Hospital, Shenzhen, China; ^3^ Department of Neurology, Changde Hospital, Xiangya School of Medicine, Central South University (The First People’s Hospital of Changde City), Changde, Hunan, China; ^4^ Hunan Provincial People’s Hospital, Changsha, Hunan, China; ^5^ Jishou University, Jishou, Hunan, China; ^6^ Department of Neurology, Xiangya Hospital, Central South University, Changsha, Hunan, China; ^7^ National Clinical Research Center for Geriatric Disorders, Xiangya Hospital, Central South University, Changsha, Hunan, China

**Keywords:** SARS-CoV-2, vaccination, anxiety, insomnia, network analysis

## Abstract

**Background:**

To explore the underlying mechanism and changes of anxiety and insomnia before and after severe acute respiratory syndrome coronavirus 2 (SARS-CoV-2) vaccination, we conducted an online cross-sectional survey.

**Methods:**

This is a cross-sectional study conducted in two phases: the first phase was from May to June 2020 (before vaccination) and the second phase was from June to August 2021 (after vaccination). In total, 2245 participants were enrolled before vaccination, and 7207 participants were enrolled after vaccination. Anxiety was measured using the Generalized Anxiety Disorder–7(GAD-7) Scale and insomnia using the Athens Insomnia Scale-8(AIS-8) Scale. Network analysis models were applied to examine the correlation between anxiety and insomnia. Furthermore, a network comparison test was performed to compare network characteristics before and after vaccination.

**Results:**

Our work showed that participants’ anxiety and insomnia scores were lower after vaccination than before vaccination. Sense of well-being during the day (AIS6) in AIS remained high both before and after vaccination. The central intensity of premature wakefulness (AIS3), feeling afraid, and functioning (physical and mental) during the day (AIS7) decreased after vaccination, and the mediation between sense of well-being during the day (AIS6) and sleeping during the day (AIS8) increased significantly.

**Limitations:**

The study was a cross-sectional survey. The numbers of participants differed much in the two groups.

**Conclusions:**

The proportion of participants experiencing anxiety and insomnia decreased significantly after SARS-CoV-2 vaccination. Network analysis identified psychosis-related symptoms as key links between anxiety and insomnia. These findings suggest that targeted interventions focusing on daytime emotional regulation could improve mental health outcomes, guiding healthcare practices during public health crises.

## 1 Introduction

The coronavirus disease 2019 (COVID-19) pandemic, caused by severe acute respiratory disease coronavirus 2 (SARS-CoV-2), has emerged as a significant global public health crisis in the 21st century (1). In response, governments worldwide implemented various preventive and isolation measures (2, 3), including remote work, social distancing, and reduced outdoor activities. Subsequent studies have revealed the profound impact of the COVID-19 pandemic on both physical and mental well-being. The prevalence of depression has surged seven fold since the outbreak (4), with 90 million new cases of anxiety disorders reported globally and a significant number of individuals experiencing insomnia (5). A nationwide multicenter cross-sectional study found that psychological and sleep problems increased during interpersonal isolation due to COVID-19 in China (6).

SARS-CoV-2 vaccination is considered a crucial strategy in combating the COVID-19 pandemic (7). On January 5, 2021, the Chinese government officially implemented a policy of providing free vaccinations to all individuals (8). Research has shown that SARS-CoV-2 vaccination not only reduces the severity of pneumonia and mortality rates (9), but also has a positive impact on the psychological well-being of the population (9, 10).

There are currently more than 400 known mental diseases, with the most common ones being depressive disorders, anxiety disorders, and various sleep disorders (11). Among these, anxiety often co-exists with insomnia, which is a common complaint among patients with anxiety and can significantly impact the course of anxiety (12). Another study found that fatigue, restlessness, and sleep disturbance are key symptoms associated with comorbid depression and anxiety (13).

Several studies have found that SARS-CoV-2 vaccination has shown improvements in symptoms of anxiety, insomnia, and depression during the COVID-19 pandemic (10, 14). However, previous studies have focused on the effects of vaccination on anxiety, depression and sleep in the population, and have not investigated the mechanisms and changes of anxiety and insomnia symptoms before and after vaccination. Moreover, traditional approaches to understanding anxiety and insomnia in psychopathology often fail to emphasize the differences between individual symptoms and their connections to typical symptoms of other syndromes (15). Network analysis is a valuable tool for understanding complex disorders and comorbidities by focusing on symptom-level interactions rather than treating symptoms in isolation. This approach identifies key “bridge symptoms” that link disorders, offering a more detailed understanding of psychopathology (16). In sleep medicine, network models have revealed that symptoms like fatigue and irritability often serve as bridges between insomnia and mental health conditions, helping to uncover mechanisms that drive comorbidities like anxiety and insomnia (17). To further investigate the correlation between anxiety and insomnia before and after vaccination, we designed a custom questionnaire to assess individuals’ anxiety and insomnia before and after SARS-CoV-2 vaccination. Additionally, we employed a network analysis model to examine the symptoms associated with anxiety and insomnia. This analysis aimed to deepen our understanding of the relationship between anxiety and insomnia, identify key symptoms that act as bridges between these conditions, and ultimately contribute to reducing the occurrence of anxiety, insomnia, and related symptoms. Consequently, this research has the potential to enhance people’s mental well-being.

## 2 Methods

### 2.1 Study samples

**Figure 1:**
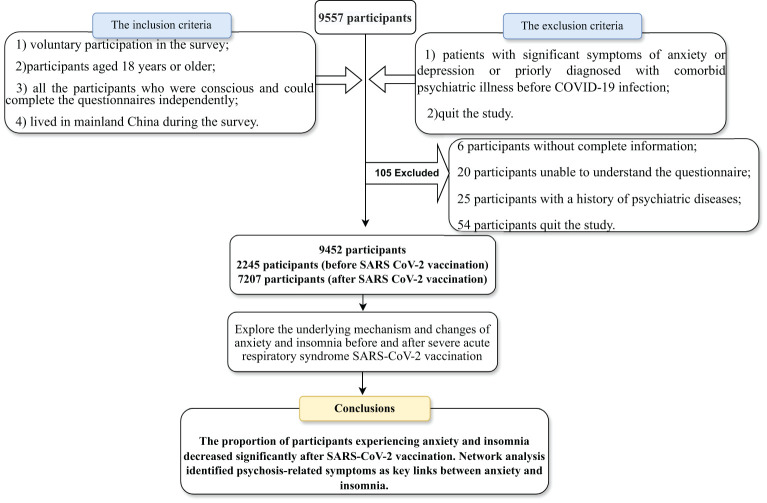
The flow chart of participant selection and exclusion.

This cross-sectional study was conducted in two phases: the first phase was from May to June 2020 (before vaccination) and the second phase was from June to August 2021 (after vaccination). Participants were extensively recruited in China to take part in the survey through the online platform “questionnaire star” including the Generalized Anxiety Disorder-7 and Athens Insomnia Scale-8. Informed consent was obtained electronically from all participants before starting data collection. Participants were free to opt out of the study at any time, even while filling in the questionnaire. Detailed information on data collection, inclusion and exclusion criteria, and demographic data are given in the **Supplementary Information**. After excluding 105 ineligible questionnaires, our final analysis included 9,452 participants. The inclusion criteria:(1) voluntary participation in the survey;(2)participants aged 18 years or older; (3) all the participants who were conscious and could complete the questionnaires independently;(4) lived in mainland China during the survey.The exclusion criteria:(1) patients with significant symptoms of anxiety or depression or priorly diagnosed with comorbid psychiatric illness before COVID-19 infection;(2)quit the study.(The flow chart describing participant selection and exclusion is shown in **Figure 1**). The Clinical Research Ethics Committee of the First People’s Hospital of Changde City approved this study (approval number: 2021-040-01).

### 2.2 Survey tools

#### 2.2.1 Generalized Anxiety Disorder–7

The Chinese version of the GAD-7 scale was used to assess participants’ anxiety. (18). The items on the GAD-7 scale, along with their corresponding reference names, are listed in **Supplementary Table S1**. Each item is rated on a scale from 0 (not at all) to 3 (almost every day), with higher scores indicating greater symptom severity. Participants with a total score of 5 or higher were considered to have anxiety, with the severity categorized as follows: 1–4 (minimal), 5–9 (mild), 10–14 (moderate), and 15 or higher (severe). The Cronbach’s alpha for this scale was 0.941, indicating excellent internal consistency.

#### 2.2.2 Athens Insomnia Scale-8

Insomnia was assessed using AIS-8 (19). Each item of AIS-8 and their corresponding reference names are provided in **Supplementary Table S1**. Of the eight items, the first five addressed participants’ sleep-related symptoms: difficulty falling asleep, difficulty maintaining sleep, and premature wakefulness. The last three items addressed the impact of any reported sleep disturbance on daytime activities: well-being, functional ability, and daytime drowsiness (20). Each item of the questionnaire could be rated from 0 to 3 (3 = severe; 2 = significant; 1 = slight; 0 = no problem/normal), with the total score ranging from 0 to 24. Participants were considered to have insomnia if the total score was ≥6 (19). Cronbach’s alpha was 0.914.

## 3 Statistical analysis

The rank sum test (Mann-Whitney U test) was used to compare changes in the total score of sleep status and anxiety before and after SARS-CoV-2 vaccination. Statistical significance was considered when P < 0.05. Network analysis was performed in terms of network estimation, network stability, and network differences.

### 3.1 Network estimation

#### 3.1.1 Network estimation

Based on network parlance, scores for each item were considered nodes, and pair-wise correlations between these scores were considered edges. Associations between each pair of nodes were determined using partial correlation analyses, after adjusting for the confounding effects of all other nodes (21). This approach ensures that the edges represent direct associations between symptoms.

To visualize the network describing the relationship between insomnia and anxiety symptoms, we first employed the Gaussian Graphical Model using the R program package “bootnet,” which estimates pairwise association parameters between all nodes. As parameter estimation can lead to false-positive associations, the relevant edges were accurately identified. In addition, the underlying the relevant edge and network structure was identified using the least absolute shrinkage and selection operator(LASSO). In the network layout, the thickness of the edges indicates the association size. Blue edges indicate negative associations, whereas red edges indicate positive associations. Network analysis evaluates the importance of a node in the network, primarily through the strength of association between a node in the network and other nodes, usually reflected by three centrality indicators: strength (sum of edge weights directly connected to the node, which indicates the importance of symptoms in the network), betweenness (number of times the shortest path between any two nodes passes through another node, which indicates the importance of symptoms connected to other symptoms), and closeness (inverse of the average, shortest path length between the node and other nodes, which indicates the degree of association between symptoms). The R package “qgraph” was used to perform centrality analysis and visualization. To assess the importance of the nodes in connecting anxiety and insomnia, bridge centrality indices of bridge strength were analyzed using the functional bridge of the R package “networktools” (version 1.2.3).

#### 3.1.2 Network stability

The accuracy and stability of the network model were examined using three procedures in order to assess the robustness of the results. In the first step, confidence intervals (CIs) were calculated using non-parametric bootstrapping to estimate the accuracy of edge-weights. By resampling and replacing the observed values, 95% confidence intervals (95% CI) can be obtained for each sampled value. In order to maintain network stability, the edge with little overlap and the 95% CI indicates that it is relatively “irreplaceable.” In the second step, the stability of centrality indices (i.e., betweenness, closeness, and strength) was evaluated using the correlation stability coefficient (CS-C) through subset bootstrapping (1000 replicates). The CS-C indicates the highest proportion of nodes that can be removed while maintaining a correlation of ≥0.7 with the original centrality measure. A CS-C > 0.5 indicates good node stability. In the third step, a bootstrapped difference test was performed to determine whether differences in edge weights or centrality indices between nodes were statistically significant.

#### 3.1.3 Network comparison

The Network Comparison Test (NCT) in R-package was used to compare the network structure before and after vaccination. NCT is a two-tailed permutation test that determines differences between two networks based on several properties such as global strength, network structure, and local edge strength. The global intensity and local edge intensity were compared between the two networks after NCT passed resampling 1000 times. Statistical significance was set at P < 0.05.

## 4 Results

### 4.1 Demographic, anxiety and insomnia characteristics

A total of 2245 participants were enrolled in the pre-vaccination cohort and 7207 in the post-vaccination cohort. The pre-vaccination cohort had a mean age of 30.47 years (SD = 8.16), and 35.2% of the participants were female. In the post-vaccination cohort, the mean age was 37.71 years (SD = 12.05), with 63.4% female participants. The geographic distribution of participants included both urban and rural areas, with 26.2% of pre-vaccination participants and 29.2% of post-vaccination participants residing in rural areas. The pre-vaccination cohort had higher anxiety scores (mean GAD-7 score: 4.25 vs.1.04, Z = 38.67, P < 0.001) and insomnia scores (mean AIS score:5.23 vs.2.37, Z = 25.55, P < 0.001) than the post-vaccination cohort. A significantly higher proportion of participants (43.7%) presented with anxiety symptoms (GAD-7 score ≥5) before vaccination than after vaccination (9.7%; χ2 = 1356.13, P < 0.001). A significantly higher proportion of participants developed insomnia symptoms (AIS score ≥6) before vaccination (43.7%) than after vaccination (16.2%; χ2 = 738.56, P = < 0.001) (**Table 1**).

**Table 1:** Demographic, anxiety and insomnia characteristics of the sample.

Characteristic	Before vaccination (N=2245)	After vaccination (N=7207)
Age [mean (SD)]	30.47 (8.16)	37.71 (12.05)
Gender (N/%)		
Male	1454 (64.8)	2638 (36.6)
Female	791 (35.2)	4569 (63.4)
Income (N/%)		
<5000	697 (31.0)	4053 (56.2)
5000-10000	980 (43.7)	2354 (32.7)
10000-20000	413 (18.4)	419 (5.8)
>20000	155 (6.9)	381 (5.3)
Degree (N/%)		
Elementary or Below	211 (9.4)	1199 (16.6)
High school	358 (15.9)	1340 (18.6)
College or Undergraduate	1470 (65.5)	4533 (62.9)
Graduate or Above	206 (9.2)	135 (1.9)
Area (N/%)		
Rural	589 (26.2)	2108 (29.2)
Urban	1656 (73.8)	5099 (70.8)
GAD-7 score [mean (SD)]	4.25 (4.54)	1.04 (2.56)
GAD-7 score group (N/%)		
Without anxiety	1265 (56.3)	6511 (90.3)
Anxiety	980 (43.7)	696 (9.7)
GAD-7 score group (N/%)		
Without depression	820 (36.5)	5477 (76.0)
Slight	445 (19.8)	1034 (14.3)
Light	671 (29.9)	573 (8.0)
Moderate	267 (11.9)	88 (1.2)
Severe	42 ( (1.9)	35 (0.5)
AIS score (mean (SD))	5.23 (4.91)	2.37 (3.23)
AIS score group (N/%)		
Without insomnia	1263 (56.3)	6039 (83.8)
Insomnia	982 (43.7)	1168 (16.2)

### 4.2 Network estimation

**Figure 2:**
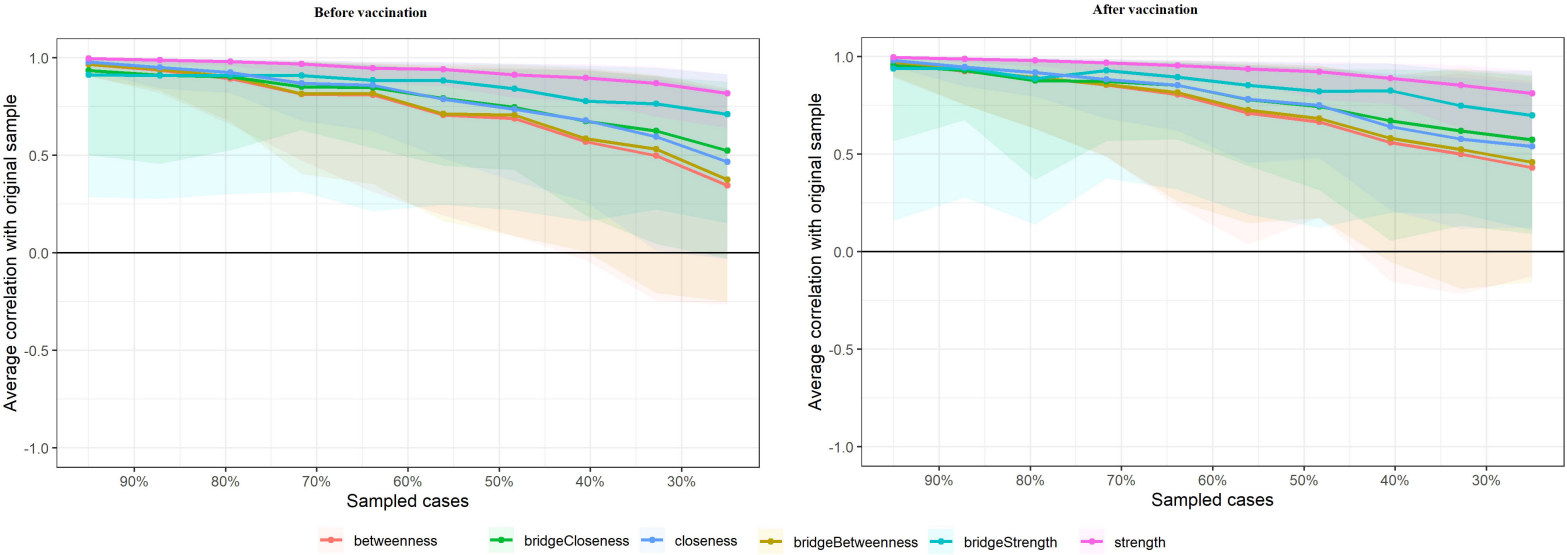
Symptom network of anxiety symptoms and insomnia during different stages of the SARS-CoV-2 vaccination. In the diagram symptom nodes with stronger connections are closer to each other. The orange nodes denote the AIS items; the blue nodes denote the GAD-7 items. The dark blue lines represent positive correlations. The edge thickness represents the strength of the association between symptom nodes.

**Figure 2** shows the network connectivity of anxiety and insomnia symptoms before and after vaccination. At both the vaccination stages, few symptoms had similar patterns of association, such as tight junctions between total sleep duration and overall sleep quality (AIS4-AIS5). Similarly, the connection between a few symptoms changed from pre- to post-vaccination. During the post-vaccination period, the connection strength between daytime physical and mental function (AIS) and the degree of daytime sleepiness (AIS7-AIS8) as well as “nervous-worry a lot” from (GAD-7) and “nervous-control worry” (GAD-7) were significantly attenuated compared with those in the pre-vaccination period. Connections between other symptoms were also attenuated to varying degrees. Notably, during the pre-vaccination period, symptoms of insomnia and anxiety were mainly connected with sense of well-being during the day as well as with restlessness (difficulty in relaxing or keeping still; AIS6-Restless, however, this connection was significantly attenuated in the post-vaccination period. Correlation matrices (**Supplementary Tables S2**, **S3**) are shown in **Supplementary Materials**.

### 4.3 Network accuracy and stability

**Figure 3:**
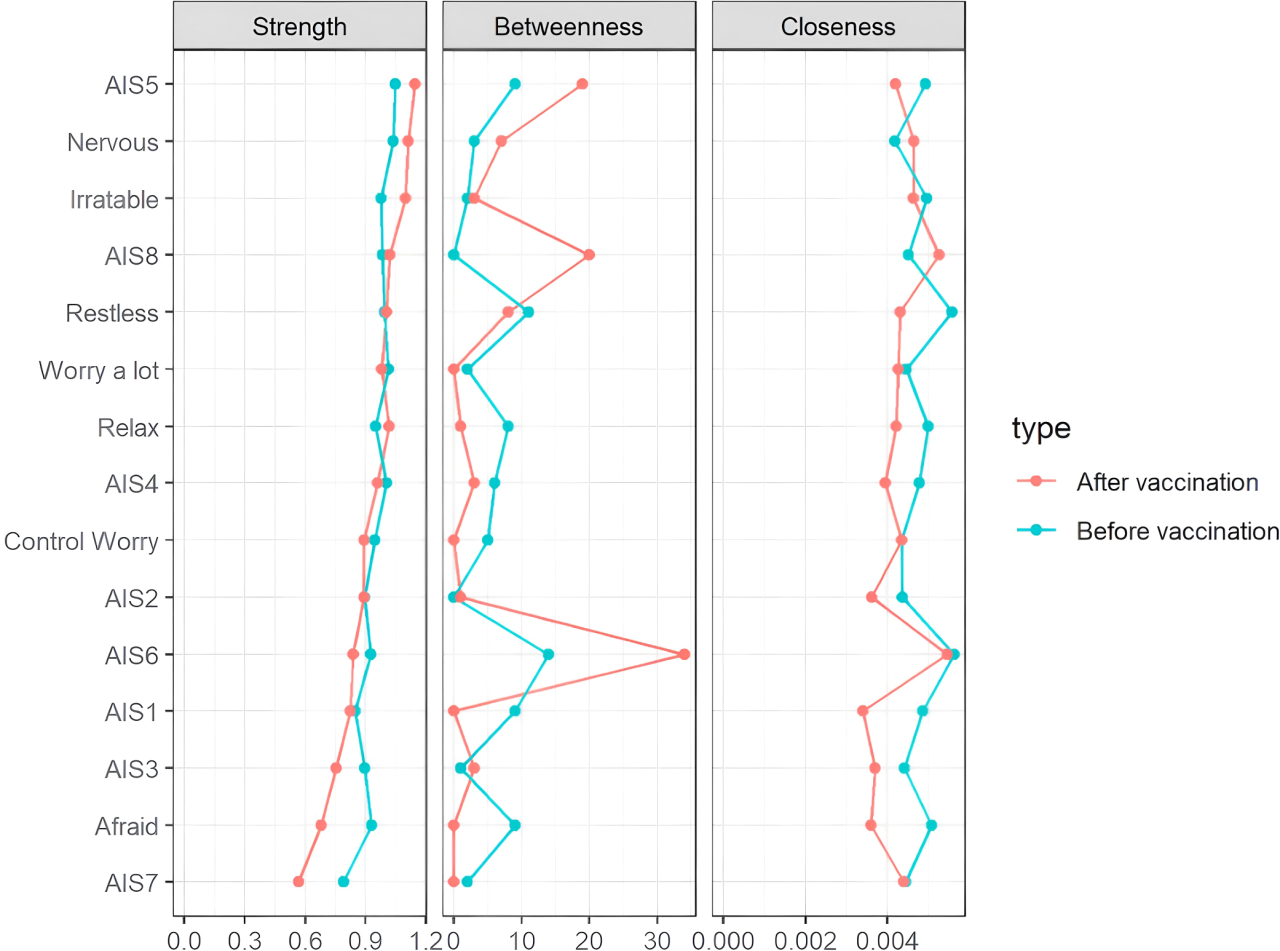
Centrality measures of all symptoms within the network at different stages of the SARS-CoV-2 vaccination. The figure shows centrality measures (i.e., strength, betweenness, and closeness) of all symptoms within the network (z-scores).

As shown in **Figure 3**, centrality indices for all symptoms are displayed for both pre- and post-vaccination periods. In the pre-vaccination network, sense of well-being during the day (AIS6) exhibited the highest strength centrality, followed by daytime sleepiness (AIS8) and daytime physical and mental function (AIS7), indicating their central roles in the symptom network. After vaccination, changes in centrality were observed, with a notable decrease in the strength centrality of premature wakefulness (AIS3), feeling afraid, and daytime function (AIS7). Additionally, the connection between sense of well-being during the day (AIS6) and daytime sleepiness (AIS8) was significantly enhanced in the post-vaccination period, highlighting these symptoms as key links in the network and suggesting their potential role in maintaining symptom associations.

As shown in **Supplementary Figure S1**, bootstrapped 95% confidence intervals (CIs) for edge weights were narrow, and the edge values were consistently higher than zero, indicating that the edge weights in the current sample were stable and consistent with the bootstrapped sample. This analysis provides assurance of the accuracy of the edge weights across the networks.

**Figure 4:**
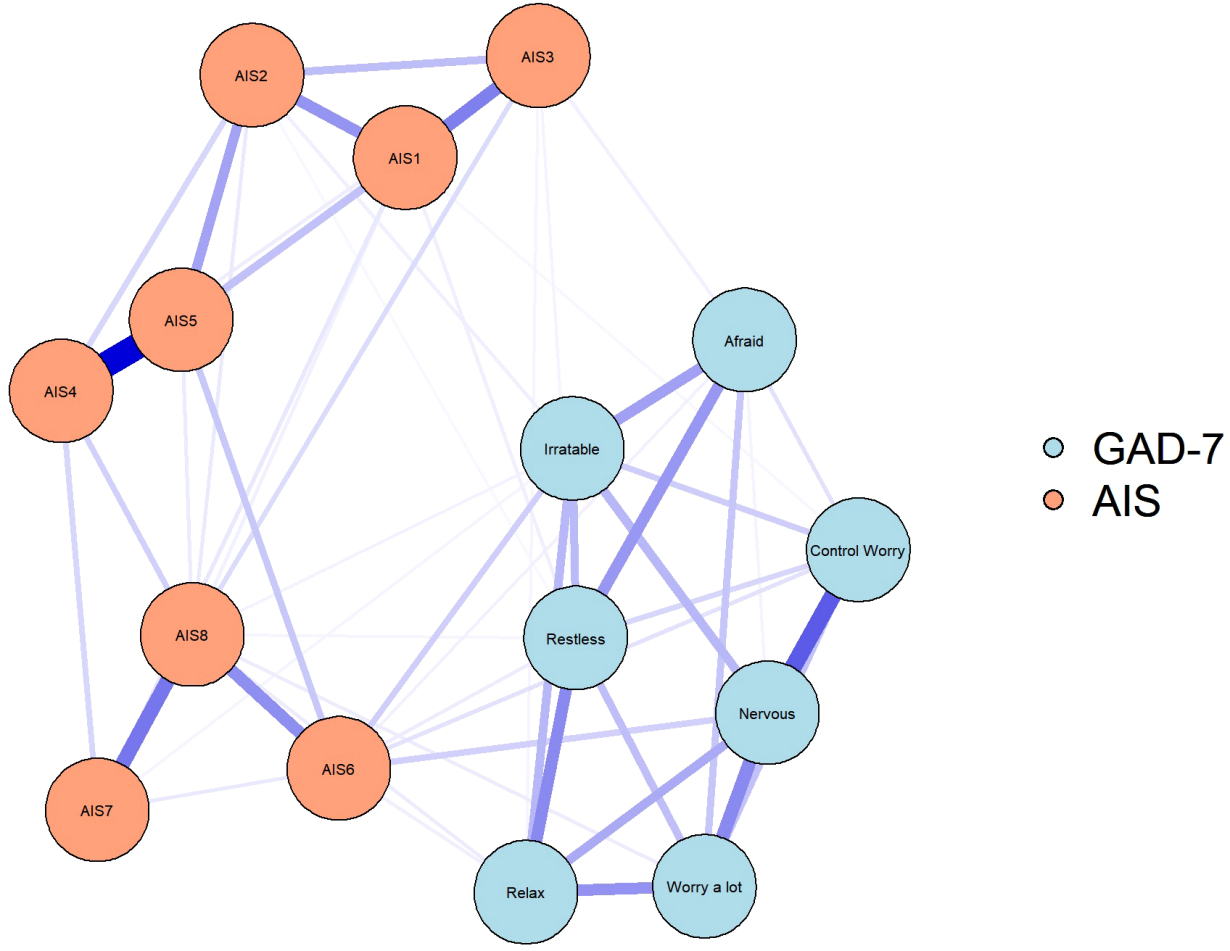
Stability of network structures. The x-axle indicates the included portion of cases, and the y-axle indicates the correlations between the original centrality indices with the estimated centrality after dropping part of the cases. Lines with different colors represent different network properties. The shades indicate the range from the 2.5th quantile to the 97.5th quantile.

**Figure 4** presents the stability of centrality indices through case-dropping subset bootstrapping. This figure shows the results of bootstrapping in which portions of the sample were systematically removed, and the stability of centrality measures was evaluated. The bridge strength of the network was extremely unstable at both stages (for both stages, CS_cor=0.7_ = 0). In addition, symptom networks at the after peak stage exhibited less stable structures, as its betweenness (CS_cor=0.7_ = 0.13), bridge betweenness (CS_cor=0.7_ = 0.13) , closeness (CS_cor=0.7_ = 0.21) and bridge closeness (CS_cor=0.7_ = 0.21) showed a CS lower than 0.25. Meanwhile, the symptom network at outbreak stage also exhibited a unstable structure with the CS of betweenness (CS_cor=0.7_ = 0.21), bridge betweenness (CS_cor=0.7_ = 0.21) , closeness (CS_cor=0.7_ = 0.28) and bridge closeness (CS_cor=0.7_ = 0.21) less than 0.25. Besides that, the symptom network exhibited a relatively stable structure with the CS of strength in the pre-vaccination period (CS_cor=0.7_ = 0.67) and in the post-vaccination period (CS_cor=0.7_ = 0.67).

### 4.4 Changes in the overall network connectivity

**Figure 5:**
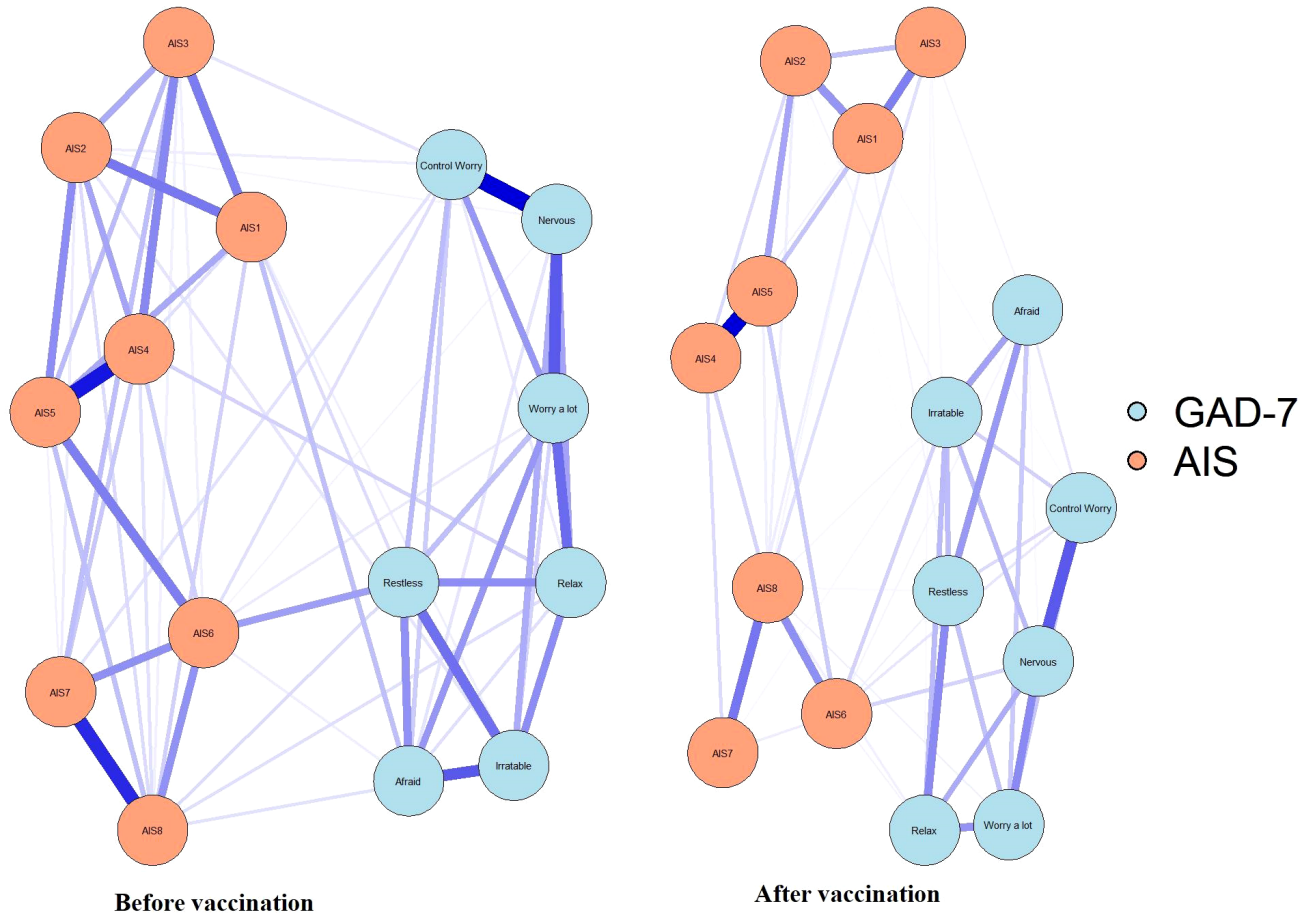
Significantly different edges between samples assessed at two different stages of the SARS-CoV-2 vaccination. The blue nodes denote the GAD-7 items, and the orange nodes denote the AIS items. Compared with before vaccination, after vaccination network showed the edge weights of AIS6-AIS4-Relax, AIS6-AIS3-Control worry, AIS6-AIS7-Control worry, and AIS6-AIS5 were significantly increased (red lines). In contrast, Nervous-AIS6 was significantly decreased (blue lines).

NCT results demonstrated that the global strength was significantly different between pre- and post-vaccination periods (S = 0.19, P < 0.001; **Figure 5**). Compared with the network before vaccination, the network after vaccination showed significantly increased edge weights of AIS6–AIS4–Relax (diff, contrast: before–after. diff = 0.05, P < 0.001, diff = 0.03, P = 0.007), AIS6–AIS3–Control worry ((diff, contrast: before–after. diff=0.03, P = 0.009, diff = 0.01, P = 0.024), AIS6–AIS7–Control worry ((diff, contrast: before–after. diff = 0.015, P = 0.037, diff = 0.02, p = 0.017), and AIS6–AIS5 (diff = 0.06, p < 0.001). In contrast, Nervous-AIS6 was significantly decreased ((diff, contrast: before–after. diff=-0.02, p=0.017). Of note, AIS6 was found to be at the center of these symptom associations and was linked to control worry from the GAD-7 through AIS7 and AIS3. In contrast, the association of nervousness with AIS6 decreased significantly during the post-vaccination period.

## 5 Discussion

The objective of this study was to explore the changes of anxiety and insomnia symptoms in the Chinese population before and after SARS-CoV-2 vaccination, and to use network analysis models to reveal their potential interaction mechanisms. To the best of our knowledge, this study presents the first network analysis of psychopathological symptoms before and after SARS-CoV-2 vaccination, providing valuable insights into the interaction between anxiety and insomnia. Demographic characteristics revealed that participants in the pre-vaccination cohort had a higher average age compared to the post-vaccination cohort. The study found that anxiety and insomnia scores were higher before vaccination and significantly decreased after vaccination, indicating that SARS-CoV-2 vaccination may alleviate anxiety and improve insomnia symptoms to some extent. Additionally, a network analysis model was employed to analyze the psychological data and identify common symptoms connecting anxiety and insomnia at different stages before and after vaccination. The results of the network analysis highlight the significance of daytime emotions as central symptoms and bridge symptoms, suggesting that targeted psychological interventions focused on enhancing daytime emotional regulation could effectively reduce the occurrence of anxiety and insomnia, thereby improving patient outcomes. Previous studies have found that depression and anxiety are associated with insomnia, and patients at a high risk of insomnia have a higher incidence of anxiety and depression than those without insomnia (22). Moreover, sleep disorders are the common symptoms of depression, and both these conditions share the same risk factors and biological basis (23). Based on the above theories, the present study determined the bridge symptoms between anxiety and insomnia to more accurately prevent these symptoms. The results revealed that the prevalence of anxiety and sleep disorders (both 43.7%) in the population was higher before vaccination than after vaccination (anxiety: 9.7%, sleep disorders: 16.2%). In a recent meta-analysis assessing the global impact of the COVID-19 pandemic, 30% of people have anxiety (24). Another meta-analysis showed the prevalence of anxiety during the pandemic was 27% (25). Compared with these, a higher prevalence of anxiety was observed in the pre-vaccination cohort of the present study. This high prevalence may be attributed to the fact that the pre-vaccination period was scheduled during the peak of the pandemic. Additionally, with the recent introduction of the vaccine, there were prevalent concerns regarding its potential side effects and efficacy (26), which were not considered within the scope of this study. Historically, during prior pandemics, vaccine hesitancy has been associated with adverse media portrayals and critical attitudes towards vaccines among influential officials (27).To further support these data, a study assessed the change in the centrality of the psychopathological symptom network after the pandemic peak (28). The findings confirmed that the psychiatric consequences of COVID-19 may continue indefinitely and may even peak later than the actual epidemic (29). In the present study, 43.7% of the population had insomnia before vaccination, which is in line with the data from a systematic review on the prevalence of sleep disorders in healthy people (18.4%–84.7%) during COVID-19 pandemic (30). However, the prevalence of anxiety and insomnia in our study decreased to 9.7% and 16.2%, respectively, after vaccination, suggesting that vaccination provided positive psychological reassurance. Participants probably believed that vaccines provide a certain protective effect against COVID-19, which may have improved their anxiety and insomnia symptoms. Furthermore, research outcomes may be influenced by temporal effects, such as the natural decline in the population’s overall anxiety levels as an epidemic subsides, which in turn can impact symptoms of insomnia. This alternative explanation has been corroborated by other studies. For example, Wang et al. conducted a nationwide survey with a sample size of 36,795 participants, revealing that while the prevalence of mental health symptoms remained elevated during the period of epidemic abatement and the resumption of work activities, a discernible downward trend was observed. (31). This suggests that environmental factors themselves may significantly contribute to the alleviation of anxiety and insomnia.

However, data were collected 2–3 months after vaccination (June to August 2021) in this study. This time window may not fully capture the long-term psychological effects of vaccination, especially since individual anxiety and insomnia symptoms may fluctuate over time (32). For instance, shortly after vaccination, individuals might experience temporary relief from anxiety and insomnia due to the sense of protection and psychological reassurance provided by vaccination. However, as time passes, these positive effects may diminish, and other stressors, such as social pressures or economic uncertainties, could resurface, potentially impacting mental health.

Moreover, the stability of anxiety and insomnia symptoms post-vaccination could change over time. Studies from a population of healthcare workers have shown that anxiety and insomnia symptoms may persist even after vaccine coverage and the pandemic eases. For example, Ding et al. highlighted in a multicenter study involving 1,412 healthcare workers that despite the majority having received public psychological education, nearly 20% continued to experience significant symptoms of anxiety or insomnia (33). This finding suggests that an individual’s psychological state is influenced by a complex interplay of factors, and vaccination alone may not suffice to comprehensively enhance mental health. While individuals may initially experience psychological relief from receiving immunization, these symptoms might re-emerge as new challenges, such as COVID-19 variants or ongoing societal stressors, arise. Thus, longer follow-up periods are necessary to assess the long-term psychological impacts of vaccination.

Network analysis showed that daytime mood was the most important symptom in the network both before and after vaccination. Accordingly, the daytime mood was used as the bridge symptom to connect anxiety and insomnia. Sleep quality may usually be judged based not only on what happens during sleep but also on mood during the waking state. Among people with insomnia, fatigue, irritability, and discomfort are more common than daytime sleepiness (34). In line with this, interventions to improve mood and functioning during the day have been found to improve self-reported assessments of sleep quality (35). A study conducted in Italy during the COVID-19 pandemic revealed that daytime sleepiness did not significantly contribute to anxiety, depression, and mood regulation processes. However, other sleep-related variables, particularly insomnia, may have a stronger association with these factors (36). For patients with psychiatric conditions, grief and worry have been found to be the most important symptoms in the anxiety and depression symptom network (37). Moreover, psychomotor symptoms including reduced exercise capacity, restlessness, and inability to relax have been shown to be the most important symptoms in the network during the COVID-19 outbreak stage (38). The results of our study showed that the intensity of each symptom in the GAD-7 scale and AIS in the post- vaccination period was lower than that in the pre-vaccination period. However, the inability to relax and daytime well-being (AIS6) showed a higher intermediary than other symptoms in both the pre- and post-vaccination periods; moreover, the intermediary of daytime drowsiness was significantly higher in the post-vaccination period. This may be attributed to the lockdown and restrictions imposed that severely limited people’s ability to engage in important social activities such as work, shopping, socialization, and leisure. In addition, these restrictions resulted in people having to face the pressure of social isolation, fear of contracting the virus, domestic violence, racial and geographical discrimination due to closures, and financial crises due to underemployment and unemployment (37). In this study, we found that the intensity, connectivity, and tightness of fear in the anxiety scale were significantly reduced after vaccination, implying that vaccination alleviated the fear of COVID-19 in the population.

This study is not without limitations. First, the interpretation of edge weights in the network analysis should be approached with caution, as they may not fully capture causal relationships between symptoms. The stability of the network, although assessed through bootstrapping, could still be influenced by sample size and composition, and missing data might affect the accuracy of the network model. Additionally, gender or age distribution differences between pre- and post-vaccination cohorts may have influenced anxiety prevalence. The notable disparity in sample size between the two participant groups in this study may also diminish the statistical power of the analysis. Furthermore, the diagnosis of anxiety and insomnia was based on self-reported measures using an online platform rather than face-to-face interviews, which may have introduced response biases. Lastly, the findings may not be generalizable beyond the Chinese population studied. In this study, data were collected 2–3 months after vaccination (June to August 2021). This time window may not fully capture the long-term psychological effects of vaccination, especially since individual anxiety and insomnia symptoms may fluctuate over time. The timing of data collection in this study may therefore affect the interpretation of the results. Future research should explore the long-term trajectory of anxiety and insomnia following vaccination to gain a more comprehensive understanding of how vaccination influences mental health over time.

In conclusion, while the cross-sectional design of this study constrains the capacity to infer causal relationships and does not entirely eliminate the potential influence of other time-related factors on the improvement of psychological states, it nonetheless offers a novel perspective on the relationship between vaccination, anxiety, and insomnia, supported by a substantial sample size and comprehensive symptom network analysis. The findings of this study suggest that SARS-CoV-2 vaccination may relieve anxiety and insomnia symptoms and highlight the critical role of daytime emotions as bridge symptoms. These results provide a foundation for future research to explore the long-term psychological benefits of vaccination, the efficacy of targeted interventions aimed at enhancing daytime emotional regulation, and the integration of mental health strategies into public health initiatives. Further studies could assess how vaccination might serve as a protective factor for mental well-being in other public health crises, contributing to more comprehensive approaches to mental health care.

## 6 Conclusion

SARS-CoV-2 vaccination may alleviate anxiety and insomnia symptoms, with daytime emotions emerging as key symptoms linking these conditions. These findings suggested that targeted psychological interventions focusing on improving daytime emotional regulation could have enhanced mental health outcomes. Incorporating such strategies into clinical practice might have helped mitigate the psychological impact of public health crises, ultimately promoting better mental well-being.

## Data Availability

The raw data supporting the conclusions of this article will be made available by the authors, without undue reservation.
