# Supplementary material for: The bridge between anxiety and insomnia symptoms among Chinese adults before and after SARS-CoV-2 vaccination: a network analysis
Source: Front Psychiatry. 2025 Jul 14;16:1604309. doi: 10.3389/fpsyt.2025.1604309 (PMC12301981; doi:10.3389/fpsyt.2025.1604309)
Supplement: Supplementary file 1 [file Supplementaryfile1.doc]

**Supplementary of figures**


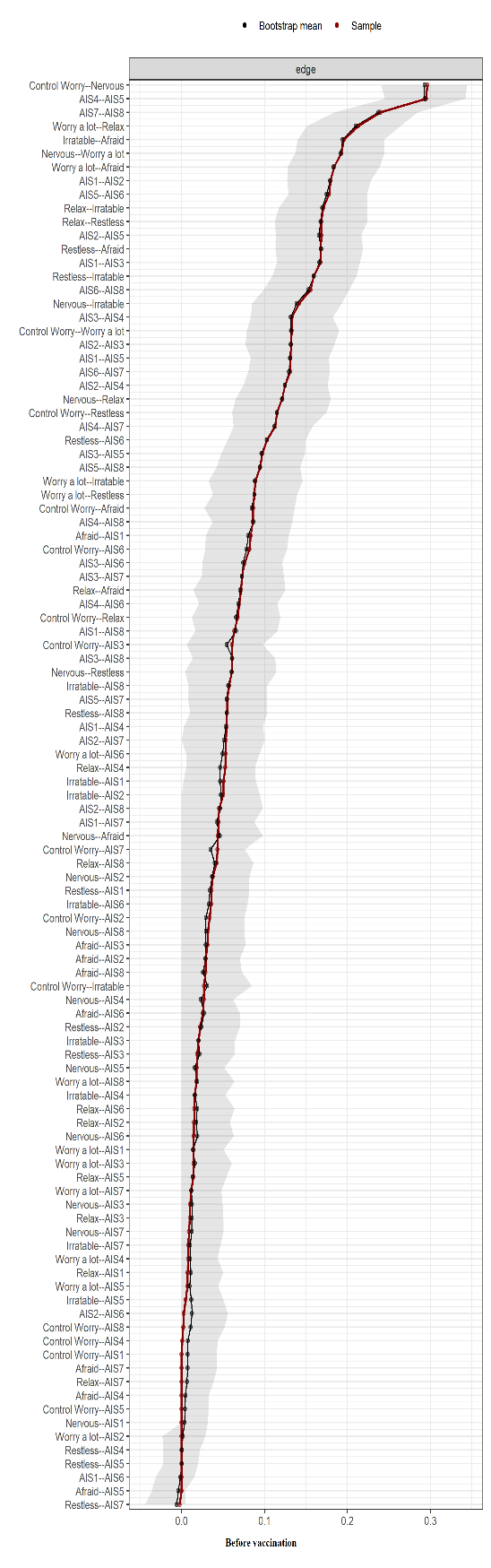

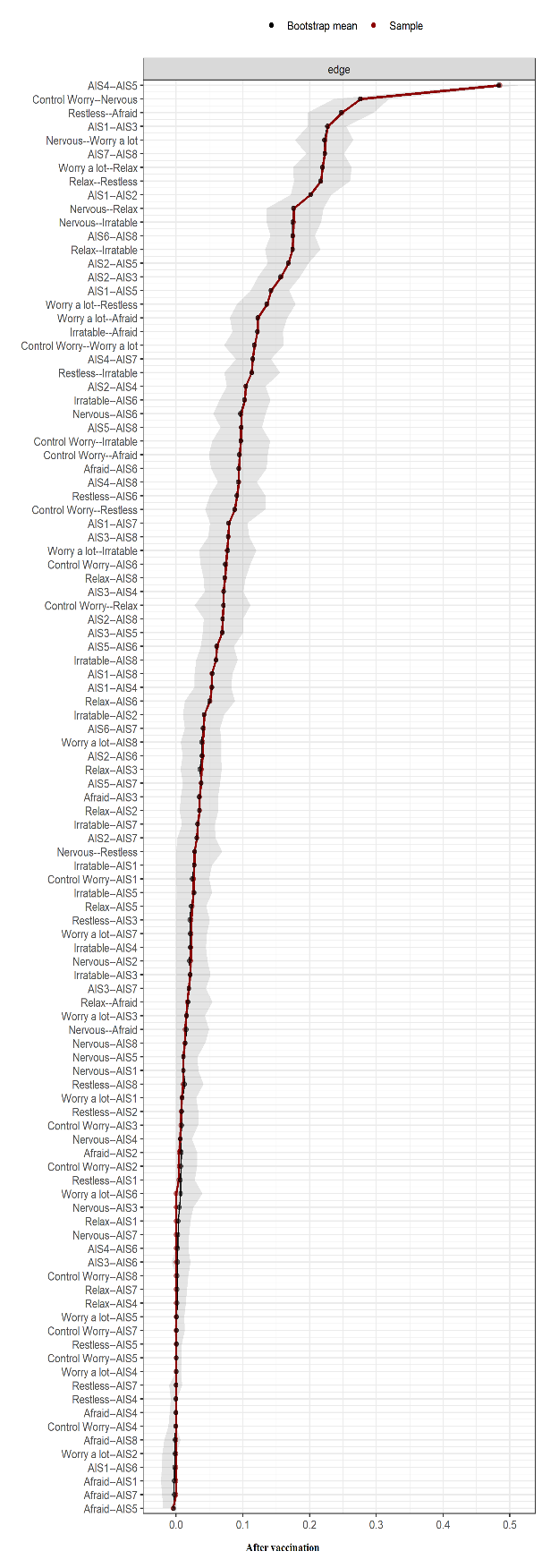


**Figure S1. Bootstrapped confidence intervals of edge weights.** The red dots indicate the values of each edge weight, ordered from the highest to the lowest values. The gray area represents the 95% Confidence Intervals of edge weights, estimated with the non-parametric bootstrap procedure. Wide intervals indicate lower stability and narrow intervals indicate higher stability.

**Supplementary of tables**

**T**able S1. Scales and items used for the assessment of psychopathology symptoms

| Scale | Symptoms | **Items** |
| --- | --- | --- |
| **GAD-7** | Nervousness | 1.Feeling nervous, anxious or on edge |
|  | Control worry | 2.Not being able to stop or control worrying |
|  | Worry a lot | 3.Worrying too much about different things |
|  | Relax | 4.Trouble relaxing |
|  | Restless | 5.Being so restless that it is hard to sit still |
|  | Irritability | 6.Becoming easily annoyed or irritable |
|  | Afraid | 7.Feeling afraid as if something awful might happen |
| **AIS-8** | AIS1 | 1.Sleep induction |
|  | AIS2 | 2. Awakenings during the night |
|  | AIS3 | 3. Final awakening earlier than desired |
|  | AIS4 | 4.Total sleeping duration |
|  | AIS5 | 5. Overall quality of sleep |
|  | AIS6 | 6. Sense of well-being during the day |
|  | AIS7 | 7. Functioning(physical and mental)during the day |
|  | AIS8 | 8. Sleeping during the day |

**Table S2**. Estimated edge weights before vaccination

|  | Control Worry | Nervous | Worry a lot | Relax | Restless | Irritable | Afraid | AIS1 | AIS2 | AIS3 | AIS4 | AIS5 | AIS6 | AIS7 | AIS8 |
| --- | --- | --- | --- | --- | --- | --- | --- | --- | --- | --- | --- | --- | --- | --- | --- |
| Control Worry | 0.00 | 0.30 | 0.13 | 0.07 | 0.12 | 0.03 | 0.09 | 0.00 | 0.03 | 0.06 | 0.00 | 0.00 | 0.08 | 0.04 | 0.00 |
| Nervous | 0.30 | 0.00 | 0.19 | 0.12 | 0.06 | 0.14 | 0.04 | 0.00 | 0.04 | 0.01 | 0.03 | 0.02 | 0.01 | 0.01 | 0.03 |
| Worry a lot | 0.13 | 0.19 | 0.00 | 0.21 | 0.09 | 0.09 | 0.18 | 0.01 | 0.00 | 0.01 | 0.01 | 0.01 | 0.05 | 0.01 | 0.02 |
| Relax | 0.07 | 0.12 | 0.21 | 0.00 | 0.17 | 0.17 | 0.07 | 0.01 | 0.02 | 0.01 | 0.05 | 0.01 | 0.02 | 0.00 | 0.04 |
| Restless | 0.12 | 0.06 | 0.09 | 0.17 | 0.00 | 0.16 | 0.17 | 0.04 | 0.02 | 0.02 | 0.00 | 0.00 | 0.10 | 0.00 | 0.05 |
| Irritable | 0.03 | 0.14 | 0.09 | 0.17 | 0.16 | 0.00 | 0.20 | 0.05 | 0.05 | 0.02 | 0.02 | 0.00 | 0.04 | 0.01 | 0.06 |
| Afraid | 0.09 | 0.04 | 0.18 | 0.07 | 0.17 | 0.20 | 0.00 | 0.08 | 0.03 | 0.03 | 0.00 | 0.00 | 0.03 | 0.00 | 0.03 |
| AIS1 | 0.00 | 0.00 | 0.01 | 0.01 | 0.04 | 0.05 | 0.08 | 0.00 | 0.18 | 0.17 | 0.05 | 0.13 | 0.00 | 0.04 | 0.06 |
| AIS2 | 0.03 | 0.04 | 0.00 | 0.02 | 0.02 | 0.05 | 0.03 | 0.18 | 0.00 | 0.13 | 0.12 | 0.17 | 0.00 | 0.05 | 0.05 |
| AIS3 | 0.06 | 0.01 | 0.01 | 0.01 | 0.02 | 0.02 | 0.03 | 0.17 | 0.13 | 0.00 | 0.13 | 0.10 | 0.08 | 0.07 | 0.06 |
| AIS4 | 0.00 | 0.03 | 0.01 | 0.05 | 0.00 | 0.02 | 0.00 | 0.05 | 0.12 | 0.13 | 0.00 | 0.29 | 0.07 | 0.11 | 0.09 |
| AIS5 | 0.00 | 0.02 | 0.01 | 0.01 | 0.00 | 0.00 | 0.00 | 0.13 | 0.17 | 0.10 | 0.29 | 0.00 | 0.18 | 0.05 | 0.09 |
| AIS6 | 0.08 | 0.01 | 0.05 | 0.02 | 0.10 | 0.04 | 0.03 | 0.00 | 0.00 | 0.08 | 0.07 | 0.18 | 0.00 | 0.13 | 0.16 |
| AIS7 | 0.04 | 0.01 | 0.01 | 0.00 | 0.00 | 0.01 | 0.00 | 0.04 | 0.05 | 0.07 | 0.11 | 0.05 | 0.13 | 0.00 | 0.24 |
| AIS8 | 0.00 | 0.03 | 0.02 | 0.04 | 0.05 | 0.06 | 0.03 | 0.06 | 0.05 | 0.06 | 0.09 | 0.09 | 0.16 | 0.24 | 0.00 |

**Table S3**. Estimated edge weights after vaccination

|  | Control Worry | Nervous | Worry a lot | Relax | Restless | Irritable | Afraid | AIS1 | AIS2 | AIS3 | AIS4 | AIS5 | AIS6 | AIS7 | AIS8 |
| --- | --- | --- | --- | --- | --- | --- | --- | --- | --- | --- | --- | --- | --- | --- | --- |
| Control Worry | 0.00 | 0.28 | 0.12 | 0.07 | 0.09 | 0.10 | 0.10 | 0.03 | 0.00 | 0.01 | 0.00 | 0.00 | 0.08 | 0.00 | 0.00 |
| Nervous | 0.28 | 0.00 | 0.22 | 0.18 | 0.03 | 0.18 | 0.01 | 0.01 | 0.02 | 0.00 | 0.01 | 0.01 | 0.10 | 0.00 | 0.01 |
| Worry a lot | 0.12 | 0.22 | 0.00 | 0.22 | 0.14 | 0.08 | 0.12 | 0.01 | 0.00 | 0.02 | 0.00 | 0.00 | 0.00 | 0.02 | 0.04 |
| Relax | 0.07 | 0.18 | 0.22 | 0.00 | 0.22 | 0.17 | 0.02 | 0.00 | 0.04 | 0.04 | 0.00 | 0.02 | 0.05 | 0.00 | 0.07 |
| Restless | 0.09 | 0.03 | 0.14 | 0.22 | 0.00 | 0.11 | 0.25 | 0.00 | 0.01 | 0.02 | 0.00 | 0.00 | 0.09 | 0.00 | 0.01 |
| Irritable | 0.10 | 0.18 | 0.08 | 0.17 | 0.11 | 0.00 | 0.12 | 0.03 | 0.04 | 0.02 | 0.02 | 0.03 | 0.10 | 0.03 | 0.06 |
| Afraid | 0.10 | 0.01 | 0.12 | 0.02 | 0.25 | 0.12 | 0.00 | 0.00 | 0.00 | 0.04 | 0.00 | 0.00 | 0.09 | 0.00 | 0.00 |
| AIS1 | 0.03 | 0.01 | 0.01 | 0.00 | 0.00 | 0.03 | 0.00 | 0.00 | 0.20 | 0.23 | 0.05 | 0.14 | 0.00 | 0.08 | 0.05 |
| AIS2 | 0.00 | 0.02 | 0.00 | 0.04 | 0.01 | 0.04 | 0.00 | 0.20 | 0.00 | 0.16 | 0.10 | 0.17 | 0.04 | 0.03 | 0.07 |
| AIS3 | 0.01 | 0.00 | 0.02 | 0.04 | 0.02 | 0.02 | 0.04 | 0.23 | 0.16 | 0.00 | 0.07 | 0.07 | 0.00 | 0.02 | 0.08 |
| AIS4 | 0.00 | 0.01 | 0.00 | 0.00 | 0.00 | 0.02 | 0.00 | 0.05 | 0.10 | 0.07 | 0.00 | 0.49 | 0.00 | 0.11 | 0.09 |
| AIS5 | 0.00 | 0.01 | 0.00 | 0.02 | 0.00 | 0.03 | 0.00 | 0.14 | 0.17 | 0.07 | 0.49 | 0.00 | 0.06 | 0.04 | 0.10 |
| AIS6 | 0.08 | 0.10 | 0.00 | 0.05 | 0.09 | 0.10 | 0.09 | 0.00 | 0.04 | 0.00 | 0.00 | 0.06 | 0.00 | 0.04 | 0.18 |
| AIS7 | 0.00 | 0.00 | 0.02 | 0.00 | 0.00 | 0.03 | 0.00 | 0.08 | 0.03 | 0.02 | 0.11 | 0.04 | 0.04 | 0.00 | 0.22 |
| AIS8 | 0.00 | 0.01 | 0.04 | 0.07 | 0.01 | 0.06 | 0.00 | 0.05 | 0.07 | 0.08 | 0.09 | 0.10 | 0.18 | 0.22 | 0.00 |
